# Reviewed article: Proença R, Nunes CCMN, Romano APM, Silva CP, Frank CHM, Moraes FS, Gonçalves FSS, Bandeira GS, Rodrigues IA, Cavalcante JR, Fonseca JLP, Silva LC, Duarte MMS, Maia MA, Mello MS, Nascimento MP, Nienov OH, Costa POM, Martins RCC, Melo SA, Ferreira VP, Casaroto VS, Freitas DRC, Almeida TVR, Garcia MHO, Barreira Filho EB. National Guidelines for Public Health Emergency Surveillance within the Brazilian Unified Health System. Epidemiol Serv Saude. 2026:35;e20260071

**DOI:** 10.1590/S2237-96222026v35e20260071.a

**Published:** 2026-10-12

**Authors:** Carlos Freitas

**Affiliations:** 1 Fundação Oswaldo Cruz, Rio de Janeiro, RJ, Brazil Fundação Oswaldo Cruz Rio de Janeiro RJ Brazil

## Peer review A

## Questionnaire

Does the manuscript contain new and significant information to justify publication? Yes

Does the Abstract (Summary) clearly and accurately describe the content of the article? No

Is the problem significant and concisely stated? Yes

Are the methods described comprehensively? Not applicable

Are the interpretations and conclusions justified by the results? Yes

Is adequate reference made to other work in the field? Yes

## Manuscript Structure

Length of article is: Adequate

Number of tables is: Adequate

Number of figures is: Too few

Please state any conflict(s) of interest that you have in relation to the review of this paper. None

Interest: Excellent

Quality: Good

Originality: Good

Overall: Good

Recommendation: Minor Revision

## Comments

O artigo aborda um tema fundamental para o SUS e absolutamente atual considerando os diferentes cenários de riscos de emergências e desastres na atualidade.

Porém, pode ser aprimorado e tornado mais didático, atingindo um amplo público de leitores e profissionais no SUS.

Sendo um artigo sobre diretrizes, a primeiro recomendação é que o item FUNDAMENTAÇÃO CONCEITUAL seja ampliado, tornando-se PRINCIPÍPIOS DO SUS E FUNDAMENTAÇÃO CONCEITUAL. Não existem diretrizes dissociadas de princípios norteadores em qualquer política pública. Em um artigo como este, é fundamental que os princípios do SUS sejam apresentados e possam se expressar de modo mais evidente nas diretrizes. Destaco a integralidade, que não aparece nas diretrizes, e a equidade, que aparece brevemente nas diretrizes e somente na comunicação de riscos. Diretrizes devem expressar claramente princípios e esta relação precisa ser melhor trabalhada no artigo.

Nas Diretrizes para a Vigilância Baseada em Indicares duas observações.

Primeiro. É importante destacar também que para além da prevalência e incidência de doenças, há indicadores que associados podem apontar para situações em que o número de casos e internações supera as capacidades dos serviços de saúde, podendo levar a desassistência, como a taxa de ocupação de leitos durante a Covid-19.

Segundo. Tomando como exemplo texto conceitual da RIPSA (https://www.ripsa.org.br/conceitos-e-criterios/) “Em termos gerais, indicadores de saúde são medidas-síntese que contêm informação relevante sobre determinados atributos e dimensões do estado de saúde da população e do desempenho do sistema de saúde. Observados em conjunto, eles devem refletir a situação sanitária de uma população e servir para a vigilância das condições de saúde e seus determinantes.

A construção de um indicador é um processo cuja complexidade pode variar desde a simples contagem direta de casos de determinada doença, até o cálculo de proporções, razões, taxas ou índices mais complexos...”

De algum modo esta conceituação deve estar melhor trabalhada e apresentada nas diretrizes, ainda que de modo mais sintético.

Nas Diretrizes para Vigilância Baseada em Eventos, complementar que há também registros de eventos pelos órgãos de proteção e defesa civil e/ou ambientais, entre outros (considerando desastres de origem natural, mas também química, nuclear, radiológica ou biológica).

Nas Diretrizes de Vigilância Baseada em Comunidades sugiro evitar o conceito de Saúde Única como está, pois é novamente abordado adiante. Sugiro uma redação como, por exemplo: situações envolvendo zoonoses e desastres ambientais (o que englobaria os de origem natural e tecnológica que é subitem da vigilância baseada em eventos).

Nas Diretrizes para Vigilância Participativa, há um documento da OMS (Putting participatory surveillance into practice) que destaca que a vigilância participativa é uma abordagem para coletar informações da comunidade a fim de monitorar tendências e que envolve uma participação proativa de membros da comunidade para relatar regularmente eventos de saúde. Do modo como está o texto aponta para uma “participação passiva”. Rever. Link para documento: https://www.who.int/news/item/16-10-2024-putting-participatory-surveillance-into-practice--new-who-publication-released#:~:text=Participatory%20surveillance%20is%20an%20approach,other%20important%20public%20health%20issues.

Ainda nestas diretrizes aparece ao final do parágrafo “...As informações coletadas eram enviadas...”, quando o certo, creio, seria: As informações coletadas podem ser enviadas.

Nas Diretrizes para Monitoramento e avaliação de risco de emergências em saúde pública importante incluir que é a primeira e fundamental informação para uma avaliação de risco (ver segundo parágrafo) é a sobre o tipo de ameaça (deslizamento, inundação, onda de calor, seca) ou perigo (agente infeccioso - tipo de vírus, bactéria ou fungo -, a substância química, o tipo de material radiológico ou nuclear, entre outros).

Nas Diretrizes das medidas de prevenção, preparação e controle há um amplo universo de ações não contempladas e que certamente vão além das 6 priorizadas no texto. Deste modo, ainda que possam priorizar algumas, não podem deixar de citar outras.

Sugiro que se tenha um parágrafo introdutório, apontando para um conjunto amplo que envolvem, de modo contínuo e circular os períodos anteriores às emergências, os de ações durante a ocorrência das mesmas (que podem variar de dias à meses e anos, como vimos no caso da pandemia), e o de ações pós fase crítica que tem como objetivo recuperação e reabilitação.

O documento Health Emergency and Disaster Risk Management Framework cita 10 componentes e funções da saúde nas emergências e desastres, que devem ser pelo menos apontadas e sinalizadas, sendo estes: 1) as políticas públicas, estratégias e legislação que norteadas pelos marcos nacionais e internacionais devem orientar uma melhor organização dos estados e municípios; 2) os mecanismos de planejamento e, principalmente coordenação, articulando os níveis nacional, estadual, municipal e local. Envolvem os planos de contingência e procedimentos operacionais já descritos, mas vão além, pois apontam fortemente para a necessidade de desenvolver mecanismos de coordenação no planejamento (e simulados); 3) os recursos humanos que vão além da formação profissional, mas que apontam para necessidade de equipe multidisciplinares, proteção e segurança para as equipes de campo, educação continuada e acreditação; 4) os recursos financeiros, envolvendo a disponibilidade de fundos para resposta e recuperação em situações de emergências; 5) a gestão da informação e do conhecimento que envolve sistemas de alerta precoce (dialogando com Salas de Situação e Centros de Operações de Emergência), proposição de agendas de pesquisas no nível nacional para as agencias financiadores e no nível estadual para as fundações de amparo às pesquisas, estratégias de incorporação e diálogo com conhecimentos locais e de povos tradicionais; 6) a comunicação de riscos, que embora já presente no texto, poderia incluir aspectos relacionados tanto as estratégias coordenadas de comunicação envolvendo diferentes mídias (um grande desafio, na época das redes sociais) e também o treinamento em comunicação de riscos para diferentes profissionais que atuam nas emergências e para os diferentes públicos, 7) a infraestrutura a logística e os serviços de saúde, o qual recomendo tomar como referência o Anexo 2 do documento da OMS (itens 7 e 8) citado acima, e incluir mais um item específico, pois já envolve muitas das ações realizadas no âmbito do Ministério da Saúde, como kits de medicamentos, vacinas, estruturas temporárias de saúde e organização temporária (adaptativa) das ações e serviços de saúde (seja APS e Vigilância durante a pandemia ou durante um desastre como o do Rio Grande do Sul em 2024).

Na Tabela 1 faltou incluir nos conceitos o de desassistência à população, que se encontra no próprio decreto citado: “Consideram-se situações de desassistência à população, (...) o evento que(...) , coloque em risco a saúde [da população] (...) por incapacidade ou insuficiência de atendimento à demanda e que extrapolem a capacidade de resposta (...) do SUS”

